# Evaluation of sealing efficacy and removal convenience of sealing materials for implant abutment screw access holes

**DOI:** 10.1186/s12903-022-02403-y

**Published:** 2022-08-25

**Authors:** Huangjun Zhou, Sixian Ye, Xingyu Lyu, Hao Feng, Min Liu, Cai Wen

**Affiliations:** 1 Department of Oral Implantology, The Affiliated Stomatological Hospital of Southwest Medical University, Luzhou, Sichuan China; 2 Luzhou Key Laboratory of Oral and Maxillofacial Reconstruction and Regeneration, The Affiliated Stomatological Hospital of Southwest Medical University, Luzhou, Sichuan China; 3 Department of Oral and Maxillofacial Surgery, The Affiliated Stomatological Hospital of Southwest Medical University, Luzhou, Sichuan China; 4 Department of Prosthodontics, The Affiliated Stomatological Hospital of Southwest Medical University, Luzhou, Sichuan China; 5 Department of VIP Dental Service, The Affiliated Stomatological Hospital of Southwest Medical University, Luzhou, Sichuan China

**Keywords:** Oral implant, Abutment, Microleakage, Sealing material, Removal convenience, Screw access hole

## Abstract

**Background:**

Sealing materials are used to fill abutment screw access holes (SAH) to prevent microleakage and protect the central screws in oral implant restoration. However, thus far, no consensus has been reached on sealing material selection. In this study, a comparison of the sealing efficacy and removal convenience of different sealing materials for cement-retained implant restoration was conducted.

**Methods:**

Various sealing materials were classified into five groups, namely, gutta-percha (GP), temporary restorative paste (TRP), vinyl polysiloxane (VPS), polytetrafluoroethylene (PTFE) tape, and onlay resin (OR), and 35 sets of analog-abutments were allocated into five groups of seven specimens. A sealing efficacy test was conducted using a modified dye-penetration method, in which a lower absorbance indicated better sealing efficacy. For the removal-convenience test, the materials were removed from each SAH after solidification, and the retrieval time was recorded.

**Results:**

On days 1 and 10, PTFE exhibited the highest absorbance value with significant differences compared to the other groups. On day 30, TRP and PTFE showed significantly higher absorbance values than GP, VPS, and OR, but no significant difference was detected between TRP and PTFE (*p* = 0.424). The absorbance values of TRP and PTFE from days 1, 10, and 30 showed significant intragroup differences, while those of the other groups did not. In terms of the removal convenience on days 1, 10, and 30, VPS achieved the best performance, followed by PTFE, OR, TRP, and GP.

**Conclusion:**

Within the limitations of this experiment, VPS and OR showed better sealing efficacy against microleakage and a more convenient removal than the other materials; thus, VPS and OR are recommended for clinical use.

## Background

Oral implants have been widely used in clinical practice because of their comfort, aesthetics, and high masticatory efficiency [1, 2]. The current mainstream implant system design is a two-piece structure composed of a lower intraosseous implant and an upper abutment. This design prevents disturbance and enables appropriate abutment direction adjustment in the intraosseous healing and prosthodontic phases, respectively. However, as different components are mechanically connected, microleakage at the interface between components cannot be completely prevented [3, 4].

The oral cavity is a complex environment; it contains saliva with a large number of electrolytes and houses 700 kinds of microorganisms [5, 6]. Saliva and these microorganisms may leak into the implant components through mechanical gaps. Bacterial colonies inside the implant could metabolise and reproduce at an appropriate body temperature and in a humid oral cavity, producing toxins and peculiar odours. Meanwhile, studies have shown that during mastication, micromotion between different components can pump bacteria and endotoxins toward the implant, abutment channel, and osseointegrating interface [3, 7–9]. Therefore, microleakage of the implant system is an important risk factor for complications, such as bone resorption, peri-implant inflammation, and central screw loosening [10, 11].

Among the oral implant system components, the abutment–implant (A–I) interface and screw access hole (SAH) could be channels for leakage [12]. Previous studies focused on the bidirectional microleakage at the A–I interface [13–15]. However, microleakage through SAH is rarely reported. The SAH cavity is conducive to oral bacterial colonisation, resulting in malodour and toxin production. In clinical practice, SAH should be sealed prior to final restoration, regardless of whether it is screw- or cement-retained. Therefore, the sealing efficacy of these materials should be comprehensively discussed. Meanwhile, when complications occur after final restoration (e.g., central screw loosening, porcelain cracking, or crown fracture) [16–18], the sealing material should be easily retrieved to facilitate the entry of a screwdriver and save chair-side operation time. To the best of our knowledge, the removal convenience characteristic of SAH sealing materials is rarely studied.

Abutment SAH sealing is a common practice in oral implantology. However, the choice of sealing materials is mostly dependent on the experience of the dentist and the availability of materials [19]. In clinical practice, various sealing materials, including gutta-percha, polytetrafluoroethylene tape, vinyl polysiloxane, zinc phosphate cement, and even cotton, are used [20–23]. SAH sealing materials should have good sealing efficacy, cost efficiency, durability, integrity, and the ability to be easily removed when necessary. However, information regarding their selection based on statistical analysis is insufficient. In this study, the sealing efficacy and removal convenience of different materials, each of which are candidates for SAH sealing and commonly used in clinical practice, are evaluated using an in vitro model of cement-retained implant crown.

## Methods

### Sample preparation

A total of 35 DAN38 implant analogs (Dentium, Korea) were connected to DAB5535HL implant abutments (Dentium, Korea) at a torque of 30 N cm; the connection type of Dentium implant system was internal. Self-curing polymethyl methacrylate (PMMA) resin (SND, China) was used to seal the interface between the implant analog and abutment to eliminate microleakage at the A–I interface. The inclusion criteria of sealing materials consider the following factors: ease of availability and reasonable economy, good sealing efficacy, and easy removal performance of materials. Thereafter, five groups of sealing materials were considered: Group A, gutta-percha (GP); Group B, temporary restorative paste (TRP); Group C, vinyl polysiloxane (VPS); Group D, polytetrafluoroethylene (PTFE) tape; and Group E, onlay resin (OR).

The sample size of the experiment was calculated by the PASS15.0 software (NCSS, LLC, Utah, USA) before the experiment. The mean (M) and standard deviation (SD) of experimental values used in the sample-size calculation were obtained by pilot experiments. The significance level was set as 5%, and the number of groups was set as five. The calculation indicated that each group required at least five samples. Therefore, 35 sets of analog abutments were allocated into five groups of seven specimens.

**Fig. 1:**
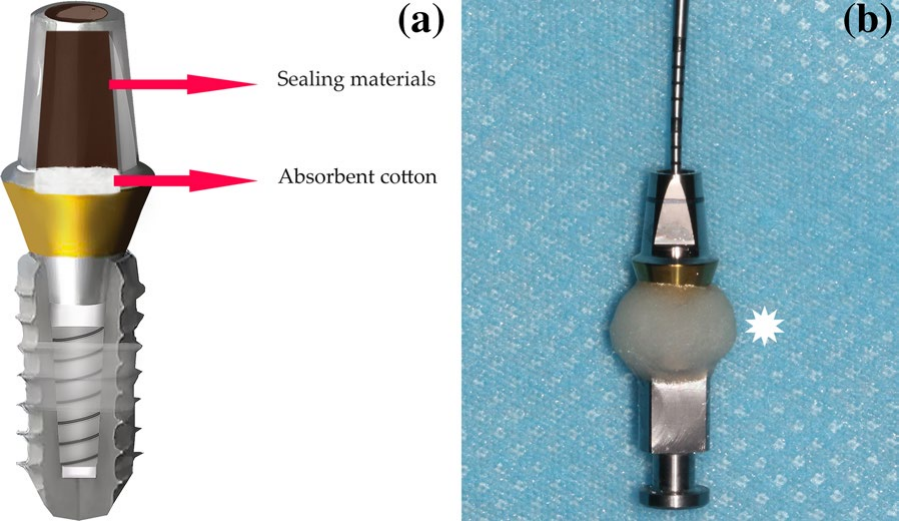
**a** Composition diagram: implant/analog body (lower part) and abutment (upper part). Inside the implant screw access hole (SAH) channel, absorbent cotton was used and compacted and then sealed with different sealing materials. **b** The abutment–implant (A–I) gap was sealed using PMMA resin (Asterisk). A scaled periodontal probe was used to ensure the same absorbent cotton thickness

**Fig. 2:**
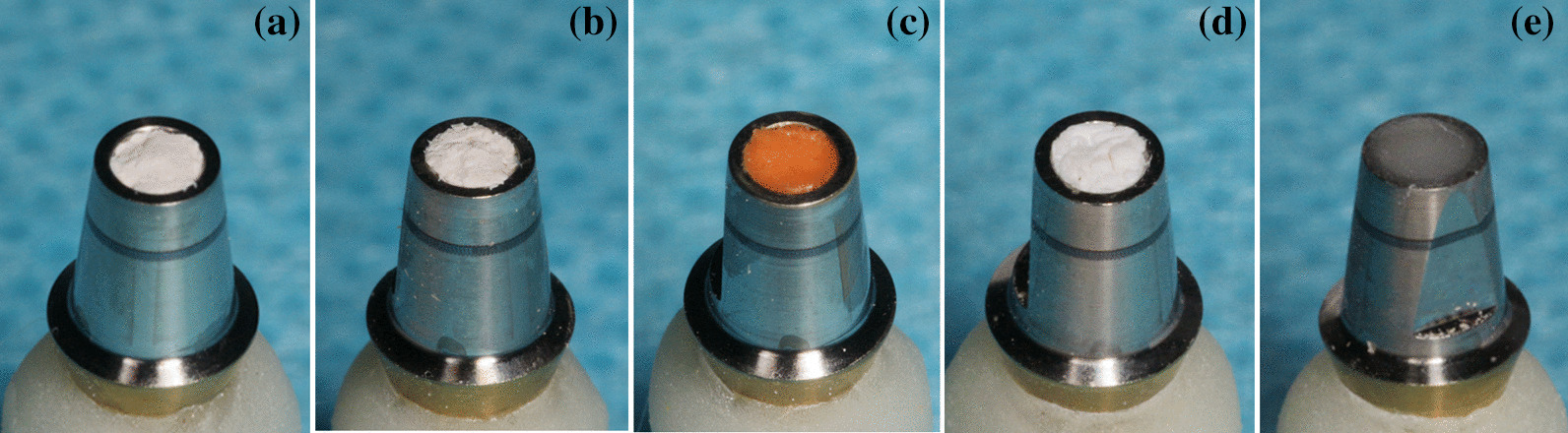
Implant abutment screw access holes were filled with sealing materials. From left to right: **A** GP, **B** TRP, **C** VPS, **D** PTFE , and **E** OR

The bottom of the abutment SAH channel was tightly filled with absorbent cotton; the filled-in cotton height was restricted to 2 mm and checked using a scaled periodontal probe. The upper residue space of the channel was sealed using different sealing materials. The filling process was standardized as follows: (a) GP (Qingpu, China) was heated over the flame of an alcohol lamp to make it soft and compressed into the SAH by a plugger; (b) TRP (META BIOMED, Korea) was directly compacted into the SAH by a plugger; (c) VPS (3 M, USA), which is the light body of silicon rubber, was slowly injected into the SAH using a mixing dispenser gun with a micromixing tip; (d) PTFE tape (Anbang, China) was twisted into a column and compacted into the SAH by a plugger; (e) Systemp OR (Ivoclar Vivadent, Liechtenstein) was kneaded into a column shape, hardened by light-curing for 2 s, inserted and compacted in the SAH, and light-cured again for 20 s. The sealed specimens were maintained at 25 °C for 24 h to ensure the complete solidification of the sealing material (Figs. 1 and 2).

### Specimen preparation

**Fig. 3:**
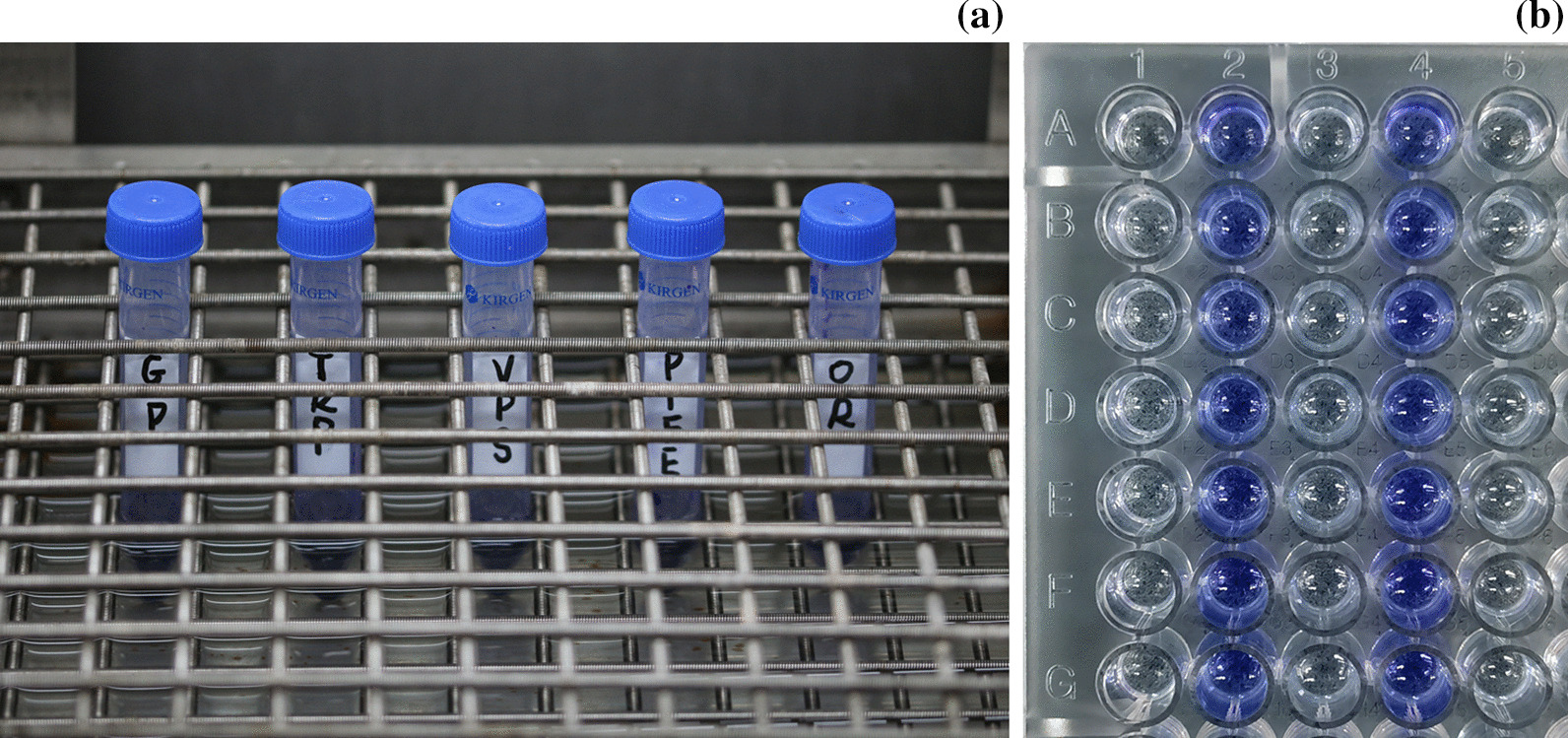
**a** Sealed analog-abutments in methylene-blue saliva were placed in a water-bath thermostat shaker. **b** 96-well plate used to measure the absorbance using a microplate reader at a wavelength of 595 nm

According to the Beer–Lambert law, as the concentration of the solution increases, its absorbance also increases, thereby enabling the analysis of microleakage by measuring the absorbance changes in the dye solution. Artificial saliva (Yuanye Bio, China) and methylene blue (Solarbio, China) were homogeneously mixed to prepare 0.1 vol% methylene-blue saliva. The sealed analog-abutment specimens were placed in centrifuge tubes and completely immersed in 4 ml methylene-blue saliva. The centrifuge tubes were then placed in a water bath thermostat shaker (Yuejin, China) at 37 °C and the speed of 40 rpm. The specimens were soaked in the shaker and shaken for 1, 10, and 30 days, respectively (Fig. 3a). Thereafter, the outer surface of the specimen was cleaned and dried using gauze rolls.

### Removal convenience test

After the required soaking and shaking time, the sealing material was completely removed from the abutment SAH by one operator (H.Z.) using a dental probe while recording the removal time. The time recording started when the operator started to remove and stopped when all materials and residues in the SAH were removed. The recorded removal time was labelled according to the grouping and soaking time, such as T_A1_, T_B10,_ and T_C30_.

### Sealing efficacy test

After the sealing material extraction, the absorbent cotton underneath was removed and placed in a 24-well plate with 95% ethyl alcohol. Methylene blue dye that infiltrated the cotton was dissolved by a horizontal decolourising shaker (Kylin-Bell, China) at 60 rpm for 2 h. The destaining solution (100 µL) from each specimen was transferred from a 24-well plate to a 96-well plate. Absorbance was measured using a microplate reader (BioTek, USA) at a wavelength of 595 nm and then recorded and labelled according to the grouping and soaking time, such as A_A1_, A_B10_, and A_C30_ (Fig. 3b).

### Statistical analysis

The experimental data were recorded using Excel (Microsoft, USA). Statistical analysis and graphics creation were performed using Statistical Product and Service Solutions 23.0 (IBM, USA) and GraphPad Prism 9 (GraphPad Software, USA). Data were presented as mean ± SD. Levene and Shapiro–Wilk tests for variance homogeneity and normal distribution were performed, respectively. One-way analysis of variance (ANOVA) and Tukey tests were used according to variance homogeneity of data; the statistical significance was set at p < 0.05.

## Results

### Sealing efficacy test

**Fig. 4:**
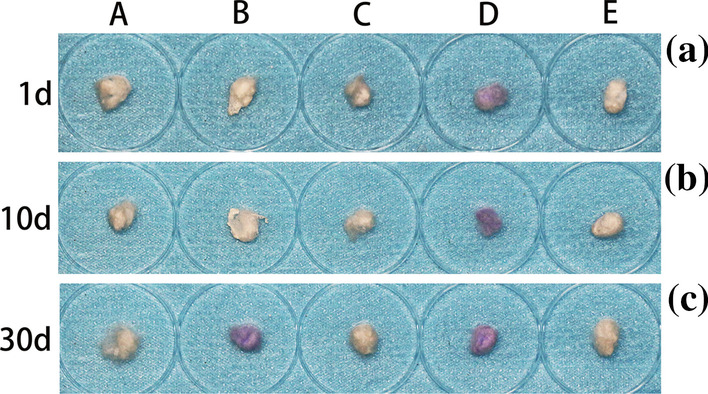
Absorbent cotton on days **a** 1, **b** 10, and **c** 30. From left to right: **A** GP, **B** TRP, **C** VPS, **D** PTFE, and **E** OR

As shown in Fig. 4, the cotton in PTFE is slightly stained on days 1 and 10. On day 30, the cottons in TRP and PTFE are stained, while those in the other groups are not.

The absorbances of the methylene blue eluate in each group on days 1, 10, and 30 are shown in Table 1. ANOVA reveals significant differences in the absorbances among the different groups on days 1, 10, and 30 (P1). The intragroup comparisons of the absorbances of TRP and PTFE on days 1, 10, and 30 show significant differences (P2), while those of the other groups do not.

**Table 1:** One-way ANOVA of absorbances of the eluate of infiltrated methylene blue on cotton, (X ± SD) × 10^−3^

Sealing material	1 d	10 d	30 d	*F2*	***P2***
GP	0.6 ± 0.5	1.9 ± 2.7	3.3 ± 1.8	3.416	0.055
TRP	1.6 ± 0.9	3.7 ± 1.7	33.7 ± 3.5	412.826	< 0.001*
VPS	1.6 ± 3.8	1.3 ± 1.3	0.9 ± 1.6	0.14	0.87
PTFE	15.7 ± 2.4	22.3 ± 0.5	31 ± 4.2	51.858	< 0.001*
OR	0.7 ± 1.7	1.1 ± 1.6	0.9 ± 2.4	0.087	0.917
F1	60.860	190.262	234.668		
P1	< 0.001*	< 0.001*	< 0.001*		

F1 and P1 are the comparisons between different groups at a same time point. F2 and P2 are the comparisons of the same group between different time points

**Fig. 5:**
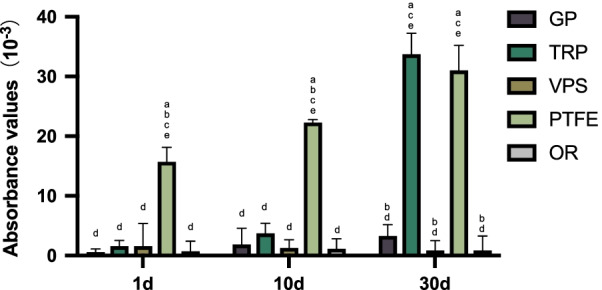
Absorbance values of eluate with different sealing materials on days 1, 10, and 30. Lowercase letters indicate statistical differences when compared with **a** GP, **b** TRP, **c** VPS, **d** PTFE, and **e** OR, *p* < 0.05

On days 1 and 10, PTFE exhibits the highest absorbance value of (15.7 ± 2.4) * 10^−3^ and (22.3 ± 0.5) * 10^−3^, respectively. The Tukey test reveals that there are significant differences among the absorbance values of the other groups (*p* < 0.001). On day 30, the absorbances of TRP (33.7 ± 3.5) * 10^−3^ and PTFE (31.0 ± 4.2) * 10^−3^ rise sharply and maintain sustained growth, respectively, both showing significantly higher results compared with GP, VPS, and OR (*p* < 0.001), but no significant difference is detected between TRP and PTFE (*p* = 0.424), (Table 1, Fig. 5).

### Removal convenience test

The index of removal time is used to evaluate removal convenience. As displayed in Table 2, for the same sealing material, the mean removal times fluctuate between days 1, 10, and 30; however, no significant difference is detected.

**Table 2:** One-way ANOVA of the removal time of different sealing materials (s)

Sealing material	1 d	10 d	30 d	*F2*	*P2*
GP	40.7 ± 7.9	43.0 ± 6.5	40.4 ± 7.4	0.26	0.774
TRP	37.0 ± 9.4	42.2 ± 7.1	43.5 ± 5.5	1.491	0.252
VPS	3.8 ± 1.3	4.0 ± 1.2	3.2 ± 1.1	0.636	0.541
PTFE	17.4 ± 4.8	19.8 ± 4.9	20.4 ± 4.11	0.828	0.453
OR	20.5 ± 5.2	20.7 ± 5.8	23.8 ± 6.2	0.719	0.5
*F1*	38.808	62.877	65.547		
*P1*	< 0.001*	< 0.001*	< 0.001*		

F1 and P1 are the comparisons between different groups at a same time point. F2 and P2 are the comparisons of the same group between different time points

**Fig. 6:**
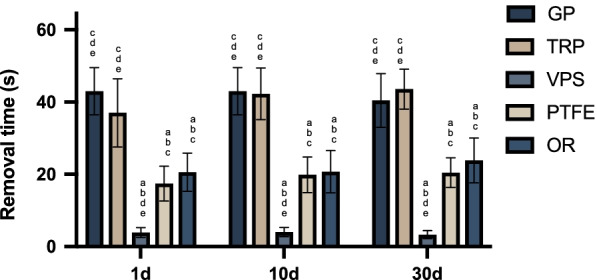
Removal times of the different sealing materials for comparison of removal convenience. Lowercase letters indicate statistical differences when compared with **a** GP, **b** TRP, **c** VPS, **d** PTFE, and **e** OR, *p* < 0.05

There are significant differences in the removal times of the different sealing materials on days 1, 10, and 30 (Table 2, Fig. 6). Furthermore, VPS has a shorter removal time than those of the other materials at each time point.

When these different sealing materials are removed on day 30, the physical states of these materials are displayed with different properties. The sealed VPS and PTFE tapes could be removed entirely in columnar and crumpled forms, respectively. Meanwhile, GP, TRP, and OR could only be removed in pieces. On day 30, VPS and TRP have the shortest and longest removal times of 3.2 ± 1.1 and 43.5 ± 5.5 s, respectively.

## Discussion

The two-piece components of the implant systems are designed to be mechanically connected, resulting in multiple interfaces which became channels for bacteria colonization, including A–I and abutment SAH. During mastication, the micromotion between components could “pump” the bacteria and saliva from the oral cavity to the interior structure of the implant and osseointegration interface through these micro-gaps [7, 24]. Although the A–I interface and SAH are both channels for microleakage, SAH is suspected as the main channel for bacterial penetration; Quirynen et al. [12] compared the microleakage of the above two channels through an in vitro bacterial experiment and indicated that the amount of bacterial infiltration at the SAH was significantly higher than that at the A–I interface. Meanwhile, selection of SAH sealing materials, which is related to microleakage, is seldom discussed. Therefore, this study investigated the effect of materials on the sealing SAH in vitro. Because material consumption is very limited, we do not consider the cost when making conclusions about the material selection. Although this study is based on cement-retained restorations, it provides guidance for the selection of sealing materials for screw-retained ones.

Although the A–I interface of the implant system is possibly the secondary leakage channel, to eliminate its influence, the complete sealing of this interface should be guaranteed. The precise fitness at the A–I interface is mainly dependent on the manufacturing precision, connection mode, and screw-tightening torque [25–29]. Studies have shown that compared with external connections, internal connections bond the abutment and implant more closely and stably, reducing the A–I interface micro-gap to decrease the infiltration of bacteria and toxins [4, 30, 31]. In addition, the connection torque is inversely proportional to the severity of microleakage [26, 32]. Carlos Larrucea et al. [26] indicated that the connection torque should be at least 20 N·cm to eliminate A–I interface microleakage. Therefore, in this experiment, strategies were used to ensure that all microleakage originated from the SAH, including using an implant system with an internal connection, analog-abutment connection with a 30 N·cm connection torque as recommended by the manufacturer, and sealing the analog-abutment interface using a self-curing PMMA resin.

Different methods have been used to evaluate implant system microleakage [33]. One method is to colonize bacteria inside the abutments before sealing and explore the inward or outward migration of bacteria, such as *Escherichia coli* and *Staphylococcus aureus* [31, 34, 35]. Another method is to evaluate the detection and quantitation of bacterial contamination with checkerboard DNA hybridisation [36, 37]. Although the microbiological method seems to have a high test sensitivity, the survival and reproduction of bacteria are easily affected by the storage environment. In an in vitro environment, the oxygenation and nutrition condition of the internal space of the implant may be affected, which could cause false-negative results owing to bacterial death. In addition, both the microbiological method and DNA hybridisation have high requirements for experimental facilities; moreover, bacterial contamination may cause false-positive results. Therefore, in this study, a modified dye penetration method was applied to quantitatively measure SAH microleakage using different sealing materials. The molecular weight of methylene blue (319.958 g/mol) is low; hence, the dye could penetrate through SAH and be absorbed by cotton at the bottom of the abutment. The methylene blue that penetrated cotton could be dissolved with ethyl alcohol; hence, absorbance values of the cotton eluate could be measured by a microplate reader. According to the Beer–Lambert law, the lower absorbance value, the lower concentration of methylene blue and the better sealing efficacy. Compared with the other method, the modified dye penetration method is simpler and more quantifiable.

The results revealed that PTFE tape, which is a common sealing material in clinical practice, has a poor sealing efficacy. PTFE microleakage was detected on day 1; it gradually increased over time. PTFE has a low free energy to resist bacterial or pigment adhesion; no chemical connections are formed after compaction because of its chemical inertness [38, 39]. Therefore, bacteria could penetrate through the micro-gap between the PTFE tape and the interface between the PTFE and abutment. PTFE tape is often used industrially to seal water pipe connections where it is wrapped in an interlocking pipe thread as a sealing medium; this is completely different compared to abutment hole sealing. PTFE tape was only compacted into the SAH and interspaces inside could not be avoided. In clinical practice, when removing PTFE tape from abutment SAHs that have been in situ for a long time, the abutments are mostly saturated with fluid and are pungent. Based on the results of this study and clinical experience, PTFE tape may not be the most appropriate choice for abutment SAH sealing.

TRP microleakage was not apparent on days 1 and 10. However, microleakage was detected on day 30, indicating that its sealing performance decreased significantly over time. The main chemical components of this temporary paste are zinc oxide, polyvinyl acetate, zinc sulfate, and ethanol. Its initial hygroexpansivity guarantees its good sealing performance within 10 days. As time increases, the hydrophobic performance of the zinc oxide material decreases owing to its loose structure, which intensifies its permeability. Therefore, although facilitated for use, TRP is not a suitable and durable option to seal the abutment SAH.

In this study, GP, VPS, and OR exhibited satisfactory sealing capacity at all time points. GP is a universal sealing material that fills root canals due to its thermoplastic and compressive properties [40, 41]. When heated and softened, GP could adapt closely to the circular lateral walls of abutment under pressure. As a common impression material, VPS, the light body of silicon rubber, can be injected to seal screw holes owing to its good flowability [42]. Meanwhile, its good liquidity, waterproofness, and low curing polymerisation shrinkage (0.15–0.2%) could reduce the gap toward the abutment wall effectively [43]. OR has a high elasticity and low polymerisation shrinkage after light curing. Additionally, it contains triclosan which has antibacterial properties. It has also been commonly used in the clinical application of implant abutment SAH sealing. Cavalcanti et al. [23] concluded that the sealing capacity of GP is superior to that of the PTFE tape, which is consistent with the results of this study. Therefore, according to the results of this study, GP, VPS, and OR have better sealing efficacies of implant abutment SAH than PTFE and TRP.

In clinical practice, dentists often use dental probes,dental excavators, endo-files, barbed broach files,and other instruments to remove the sealing material prior to unscrewing the implant abutment in the process of retrieving implant restoration. Different sealing materials have different removal features, which significantly affect chair-side time. Within the limitation of placing absorbent cotton above the screw head, which is slightly different from clinical practice, the results indicated that the removal of VPS was the fastest, followed by those of the PTFE tape and OR, whereas GP and TRP were the most time-consuming. The cured VPS was elastic, which allowed intact removal. PTFE tape can be easily removed owing to its hydrophobic lubrication and incompactness. However, GP, TRP, and OR could only be removed in several pieces because they became hard and brittle after complete solidification. In fact, chair-side time increases if these sealing materials cannot be completely removed in one attempt. Furthermore, if the sealing material disintegrates easily, improper handling may generate residue inside the internal screw thread that affects the unscrewing of the centre screw and passive position when tightening, causing serious complications, such as central screw fracture.

Except for the single use of sealing material, it has been suggested that two or more materials can be combined to seal the SAH. Nascimento et al.[22] combined PTFE, GP, composite resin, and cotton in pairs to seal the SAH. It was found that the combination containing composite resin and GP has a lower microleakage. However, sealing by combining two or more materials would increase the interfaces between materials, and that protocol design was not adopted in this study.

### Limitations

First, as an in vitro experiment, the experimental model could not be completely consistent with the situation in vivo. On one hand, after the final restoration, the abutment in patient’s mouth is covered by crowns, which may limit bacterial microleakage through SAH compared to in vitro experiments. On the other hand, after crown restoration, deformation and micro-movement of the interface during cyclic loading may quicken the pumping effect of bacteria or saliva fluids. Because the influence of crown restoration on leakage is uncertain, and the simplified model without crown restoration does not affect the comparison of material sealing efficacy, therefore, the cyclic loading of the crown was not conducted. Second, owing to the near-constant mouth temperatures, the thermocycling test with a large temperature variation—which simulated aging effects—may not be appropriate. Indubitably, SAH sealing material may need to remain in situ for decades, and its long-term degradation and proper aging model construction continue to be points of interests and targets for future studies. Therefore, conclusions are only be drawn based on the short-term performance of the sealing materials.

## Conclusions

In this experiment, the short-term sealing performance and removal convenience of sealing materials for implant abutment SAH were compared quantitatively. Within the limitations inherent to this in vitro study, the following conclusions were drawn:

1. VPS, OR, and GP showed better sealing properties than TRP and PTFE when used to seal SAH.
2. The removal convenience of VPS, OR, and PTFE tape was better than those of TRP and GP.

Therefore, VPS and OR are the most recommended sealing materials for clinical implant SAH fillings, as they could reduce microleakage and improve clinical operability.

## Data Availability

The data presented in this study are available on request from the corresponding author.

## Abbreviations

A–I: Abutment–implant
SAH: Screw access hole
PMMA: Polymethyl methacrylate
GP: Gutta-percha
TRP: Temporary restorative paste
VPS: Vinyl polysiloxane
PTFE: Polytetrafluoroethylene
OR: Onlay resin
ANOVA: One-way analysis of variance
